# Convergent Evolution of Copy Number Alterations in Multi-Centric Hepatocellular Carcinoma

**DOI:** 10.1038/s41598-019-40843-9

**Published:** 2019-03-14

**Authors:** Carolin Lackner, Luca Quagliata, William Cross, Sebastian Ribi, Karl Heinimann, Viola Paradiso, Cristina Quintavalle, Monika Kovacova, Daniel Baumhoer, Salvatore Piscuoglio, Luigi Terracciano, Michal Kovac

**Affiliations:** 1 Institute of Pathology, Auenbruggerplatz 25, 8036 Graz, Austria; 2 Institute of Pathology, University Hospital Basel and University of Basel, Schoenbeinstrasse 40, 4056 Basel, Switzerland; 3 Department of Tumour Biology, Barts Cancer Institute, Queen Mary University London, London, UK; 4 Medical Genetics and Research Group Human Genomics, University Hospital Basel and University of Basel, Schoenbeinstrasse 40, 4031 Basel, Switzerland; 5 The Institute of Mathematics and Physics, Faculty of Mechanical Engineering, Slovak University of Technology, 84248 Bratislava, Slovak Republic; 6 Visceral Surgery Research Laboratory, Clarunis, Department of Biomedicine, University of Basel, Basel, Switzerland

## Abstract

In the recent years, new molecular methods have been proposed to discriminate multicentric hepatocellular carcinomas (HCC) from intrahepatic metastases. Some of these methods utilize sequencing data to assess similarities between cancer genomes, whilst other achieved the same results with transcriptome and methylome data. Here, we attempt to classify two HCC patients with multi-centric disease using the recall-rates of somatic mutations but find that difficult because their tumors share some chromosome-scale copy-number alterations (CNAs) but little-to-no single-nucleotide variants. To resolve the apparent conundrum, we apply a phasing strategy to test if those shared CNAs are identical by descent. Our findings suggest that the conflicting alterations occur on different homologous chromosomes, which argues for multi-centric origin of respective HCCs.

## Introduction

Hepatocellular carcinomas (HCC) with intra-hepatic metastases (IM) are profoundly different in their development and clinical outcome from multi-centric tumors^1^. The clinical discrimination between these subtypes has historically been challenging and is usually based on tumor location, blood vessel involvement of the primary tumor, and/or hemodynamics in CT/MRI imaging before resection^2^. Once tumors are resected, pathological evaluation is performed to discriminate between the IMs and MCs. However, there is no consensus at present on how to discriminate the two and it is advised to use complementary molecular tests to reduce the uncertainty. For example, in a recent study Furuta *et al*. showed similarities between genomes of multi-centric tumors using recall-rates of somatic alterations^3^, while others achieved the same with transcriptome and methylome data^4,5^. In this study, we extend Furuta’s strategy by accounting for convergent evolution of copy-number alterations (CNA) in multi-centric tumors that can, in certain situations, complicate data interpretation and clinical reasoning.

## Results

**Figure 1:**
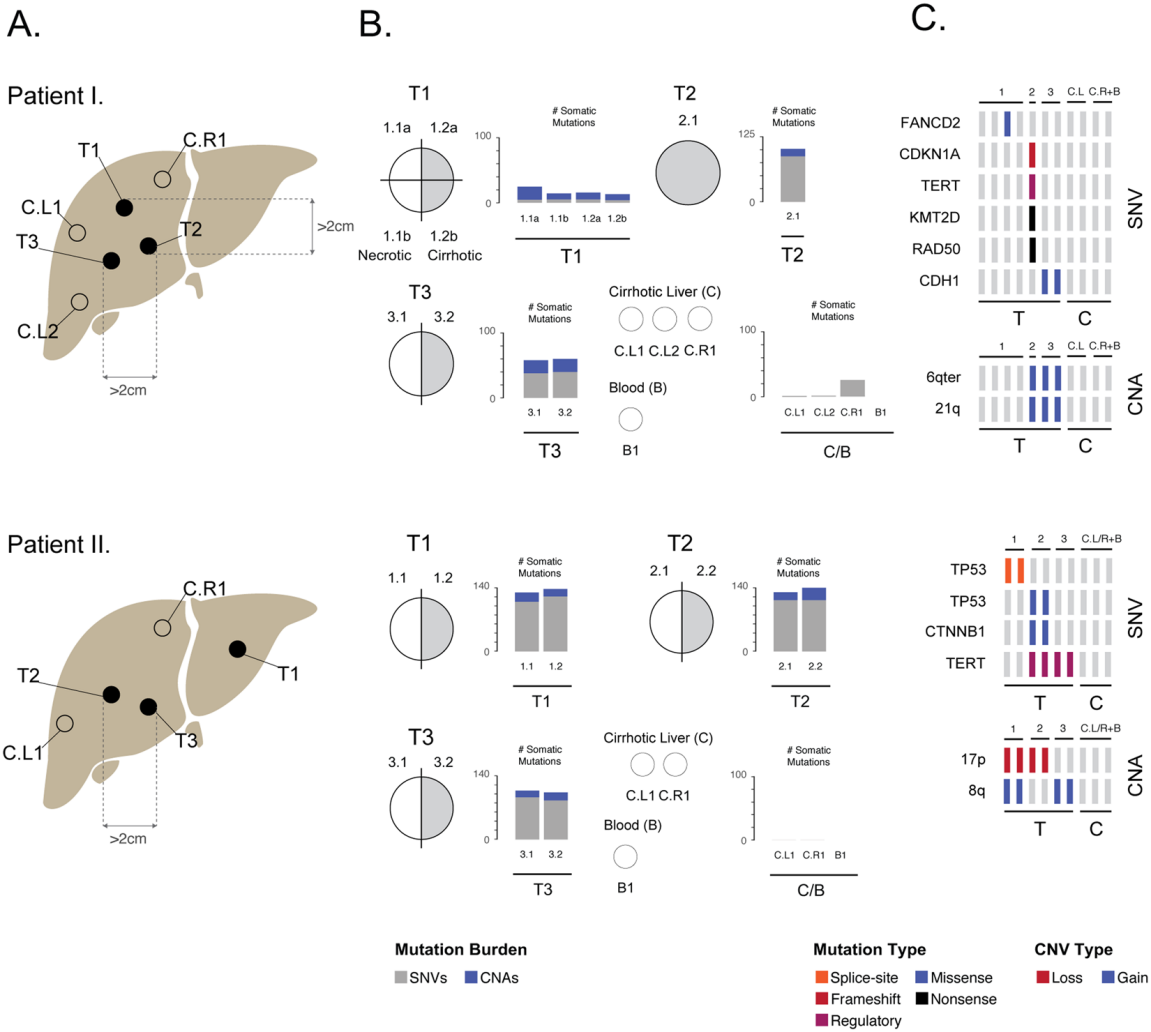
Multi-region exome sequencing of multi-centric hepatocellular carcinoma. (**A**,**B**) Sampling scheme and mutation burden of tumor regions (grey: single-nucleotide variants, blue: indels). (**C**) Cancer driver mutations. Abbreviation: T: tumor, L: left, R: right, (**C**) control, matched normal tissue/blood, CNA: copy-number alteration, SNV: single-nucleotide variant.

**Figure 2:**
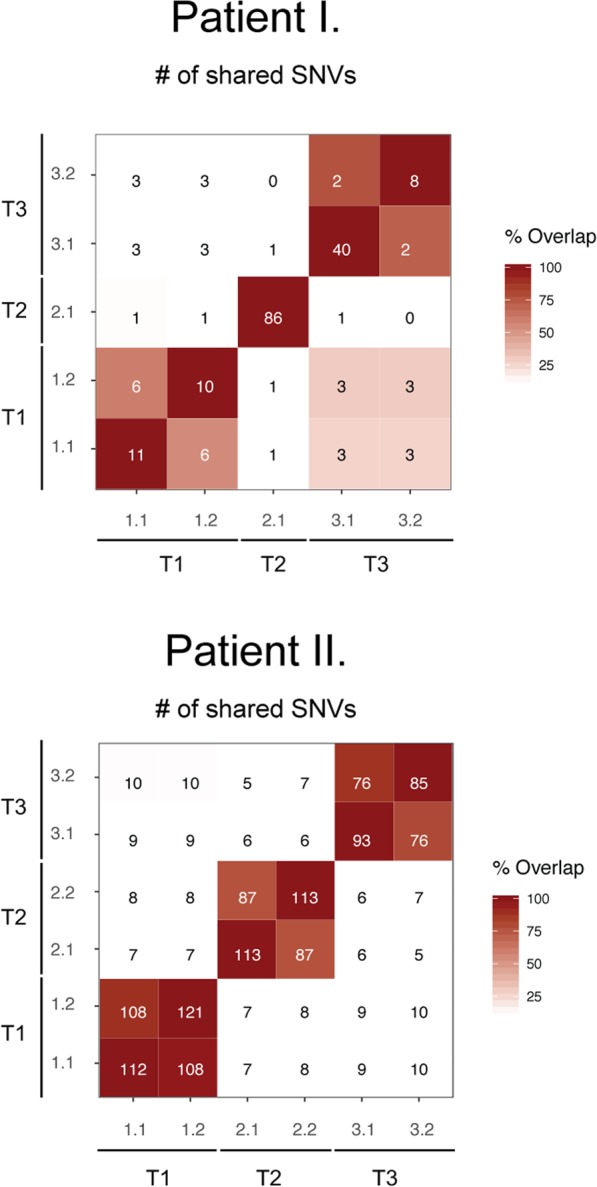
Pairwise comparison of mutation burden across multi-centric tumors and corresponding tumor regions. For each tuple, number of shared mutations are indicated, whilst color intensity corresponds to their overlap (in %).

We generated testing data from multiple-regions of chemotherapy-naïve multi-centric HCCs of two HBV/HCV-negative patients (Table 1). In each patient, somatic mutations were identified from multi-region exome sequencing data from three synchronous tumors (Fig. 1A). Prior to the analysis, sequencing reads were mapped onto the human genome hs37d5 and GATK haplotype caller was used to identify single-nucleotide (SNV) mutations and indels simultaneously. On average, 42.8/21.0 somatic SNVs and 106/15.3 indels were detected per tumor region per patient (Fig. 1B, Supplementary Table 2), of which six and four variants per patient were cancer driver mutations (Fig. 1C). Pairwise comparison of somatic SNVs across all regions of a given tumor yielded average recall rates of 65% and 85% per patient, while across different tumors 10.9% and 7.3% (Fig. 2; Supplementary Tables 3–6).

**Table 1:** Clinical description of HCC patients.

Patient	Age at diagnosis	Liver disease	HCC positions according to the radiology report	HCC positions according to the pathology report	Histological classification	TNM	Macroscopic vascular invasion	Microscopic vascular invasion	Grade	Inclusions in tumor cells	Other features
1	58	alcohol-related cirrhosis	right lobe, segment 7, only 1 nodule	3 nodules in the right lobe, none of the nodules within 2 cm distance of the other	trabecular and pseduglandular	pT2NxMx	no	no	G2	no	bile production
2	57	alcohol-related cirrhosis	left lobe, only 1 nodule	1 nodule in the left lobe, 4 nodules in the right lobe, none of the nodules within 2 cm distance of the other	trabecular and pseduglandular	pT2NxMx	no	no	G1	no	bile production

We therefore ensured that there was not a significant enrichment of false positive variants in our analysis by replicating 21/23 (91%) selected somatic mutations and re-assessing hematoxylin-eosin stains of tumor tissues to ensure that they contained at least 80% of tumor cells (Supplementary Table 7). We then undertook a down-sampling approach to equalize read depth across all tumor regions and repeated the analysis with additional cancer-specialized calling algorithms, including MuTect2^6^ (i.e. GATK4 implementation of MuTect) and Strelka^7^. Nevertheless, read-depth equalizing or the use of different variant calling algorithms did not improve the recall rates and a gap between the degree of inter- and intra-tumor heterogeneity remained.

**Figure 3:**
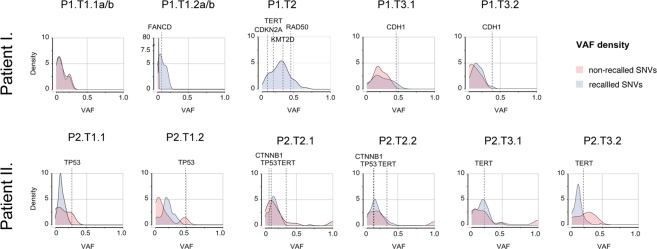
Variant allele frequency distributions of somatic mutations.

**Figure 4:**
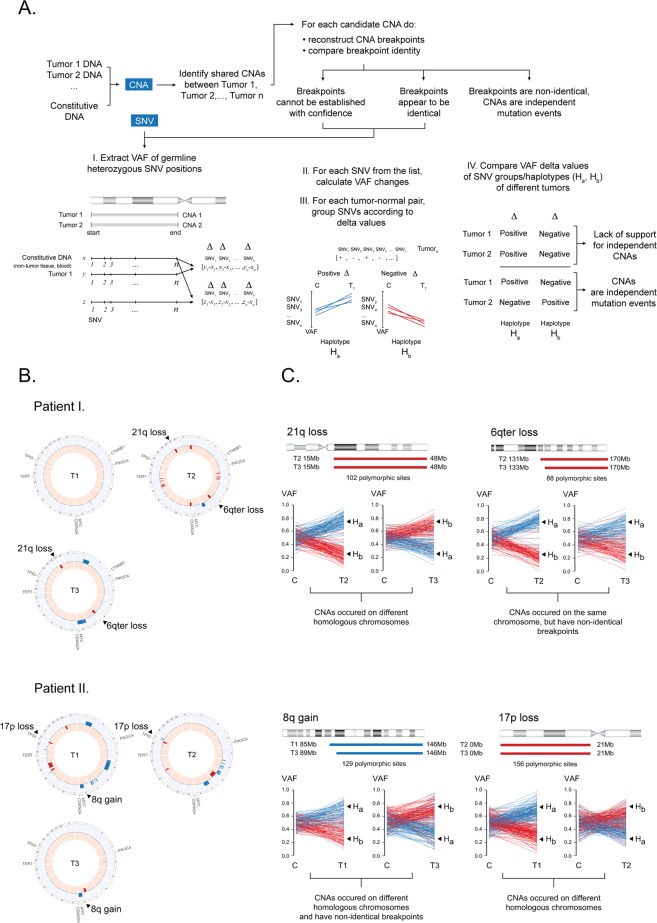
Copy-number alterations (CNAs) and chromosome phasing. (**A**) An algorithmic overview. (**B**) Copy-number profile of multi-centric HCCs. Candidate CNAs are indicated by arrow. Chromosomal gains are blue, losses are red. (**C**) Haplotype inference using polymorphic sites and their respective allelic frequency changes. Abbreviation: (**C**) control; blood sample or non-tumor tissue, SNV: single-nucleotide variant, T: tumor, H: haplotype.

Most somatic SNVs occurred at low allelic frequencies irrespectively of whether they were recalled in different regions of the same tumor or not (Fig. 3; Supplementary Tables 8–9). Since cancer driver mutations were no exception and thus no clearly clonal candidate was found, we wondered if some other type of mutations could help us to classify multi-centric tumors of our patients. For example, there were four possibly identical copy-number alterations (CNA; Fig. 4) occurring in two tumors of each patient. To determine if these CNAs were not identical by descent, we used a combination of breakpoint analysis and chromosome phasing. The specific phasing algorithm is detailed in material and methods and on a Fig. 4 and is, at heart, a Welch’s two sample t-test that compares allele-frequency changes at polymorphic sites of candidate CNAs of tumor samples and a tumor-free tissue, on the assumption that allele frequencies for each chromosomal haplotype are random variables with mean μ and variance σ^2^. Our null hypothesis was that CNAs occurred at the same homologous chromosome in different tumors. After reviewing each case, the null hypothesis was rejected on a significance level of 0.05 for cases of CNAs of chromosome 21q, 8q and 17p, whilst accepted for the case of 6q. In this case, however, we used the breakpoint information to argue that 6qter alterations were independent mutation events.

## Discussion

In this study, we attempted to classify multi-centric hepatocellular carcinomas using the information that may be not be available for strategies that either compare tumor morphology or mutation burden. Especially, the use of the latter as proposed by Furuta *et al*. can be sometimes misleading because the assignment of single or multiple origin category is possible only for tumors which share most or no somatic mutations, but otherwise there is no clear threshold to distinguish them. Given that cancer driver mutations can occur in cells that do not undergo malignant transformation^8,9^, it could well be that multi-centric tumors originating independently can be difficult to distinguish from IMs that have arisen very early^10^. It is also possible that these tumors behave differently in development and clinical outcome from IMs that originated late in progression and are classified as true IMs. Thus, if disease prognosis and/or clinical classification of IMs is the end-point of an analysis, molecular strategies utilizing somatic mutations may not be precise enough to distinguish the differing clinical categories. Whilst our study illustrates how refinements of the existing methods can be used for better data interpretation, it also outlines some of the greater limitations that must be overcome before molecular strategies are implemented clinically.

## Methods

### Patients and samples

The study was approved by the ethical committee of the Medical University of Graz (ethical approval 26-170-ex-13/14) on 20^th^ January 2014. All methods were performed according to the relevant guidelines and regulations. Informed consent for study participation was obtained from both patients. DNA was extracted from six fresh-frozen tumors and each tumor was sampled at least twice. Pathological examinations of tumor samples were performed by experienced pathologists to ensure that each biopsy contained at least 80% of tumor cells. DNA derived of 20 samples was subjected to Illumina HiSeq. 2000 pair-end sequencing, including six tumor-free (cirrhotic) liver parenchyma and two blood samples.

### Illumina sequencing

Whole exome sequencing (WES) was performed using the Illumina HiSeq. 2000 platform after short insert ~400 bp libraries were constructed, flow cells prepared, and clusters generated. For WES target DNA was enriched by bait capture (Agilent SureSelect ver. 4) and short-insert libraries were sequenced to average depth of 118X (IQR 56, Supplementary Table 1). Sequencing data are freely available from the authors after a reasonable request.

### Semiconductor sequencing

Ion torrent sequencing was selected for technical replication of somatic variants. Used in conjunction with the AmpliSeq Library Kit 2.0, the Ion AmpliSeq™ Comprehensive Cancer Panel was selected to capture exons of 409 genes from the Cancer Gene Census database and the resulting libraries were ran on the Ion PGM™ Sequencer. The raw reads were processed using the Ion Reporter software with recommended settings.

### Sanger sequencing

Following PCR amplification, Sanger sequencing was used to detect TERT promoter mutations g.5:1295228 G > A/T and g.5:1295250 G > A. The primer sequences were 5′-CCAGGGCTTCCCACGTGC-3′ for the forward primer and 5′-ACTGGGGACCCGGGCACC-3′ for the reverse primer.

### Variant Detection and Filtering

Raw sequencing reads were quality-checked (fastqc ver. 0.11.7), adapter-trimmed, duplicate-removed (Picard tools ver. 2.9) and mapped onto the hs37d5 version of the human genome (BWA ver. 0.7). The GATK pipeline (ver. 3.7) was used to perform base-quality score recalibration and variant calling. Specifically, we used GATK haplotype caller algorithm with standard settings, followed by Variant Quality Score Recalibration (VQSR) for single-base substitution identification and the Scalpel algorithm (ver. 0.5.3) for indels. Variant files containing too few variants were specifically filtered (QD > 10.0, MQ > 40.0, FS < 30.0, SOR < 3.0, MQRankSum > −12.5, ReadPosRankSum > −8.0) to extract high-quality variants. We used the human genome assembly hs37d5 and 2017 versions of ANNOVAR databases to annotate variants. Germline or somatic origin of the variants and indels were determined based on their presence or absence in the matched tumour-free tissue.

We applied the following exclusion filters to somatic variants: (i) presence in a segmental duplication region; (ii) variant present in any read from paired normal sample; (iii) fewer than ten reads in total at the variant site in the normal sample; (iv) fewer than eight reads in total in the tumor; (v) fewer three variant reads in the tumor; variant allele frequency <3% in the tumor; and (vi) presence of variant in the Exome Aggregation Consortium dataset (release 22.6.2017) at a frequency >2%. Variants identified in constitutional DNA from any of the other local, non-cancer sequencing project at a frequency of 5% (for example, 29 million variants across 284 samples from the Oxford-Illumina WGS500 consortium) were discarded as being more likely due to systematic error in our pipeline than genuine somatic mutations.

### Copy Number Calling

Nexus Copy Number Discovery software (ver. 9.0) was used to identify CNAs from sequencing data and for independent validation of these alterations with Affymetrix Oncoscan arrays. The raw array data were processed with Affymetrix ChaS software, (ver. 3.2) and imported as segmental reports into the software. Only CNAs larger than 50Kb with a minimum support of 21 probes were considered for analysis.

### Chromosome Phasing

Allele frequencies (AF) of polymorphic sites of candidate CNAs were extracted and AF differences between tumors and a normal tissue were calculated. For each case, corresponding chromosomal haplotypes were inferred from SNVs with AFs shifted in the same direction (i.e. either positively or negatively). Welch two sample t-test was then used to compare AF values of respective haplotypes across different tumors on the assumption that AF observations are random variables with mean ${\mu }$ and variance *σ*^2^.

## Supplementary information

Supplementary Information
