# Supplementary material for: Convergent Evolution of Copy Number Alterations in Multi-Centric Hepatocellular Carcinoma
Source: Sci Rep. 2019 Mar 14;9:4611. doi: 10.1038/s41598-019-40843-9 (PMC6418287; doi:10.1038/s41598-019-40843-9)
Supplement: Supplementary file 1 — Supplementary Information [file 41598_2019_40843_MOESM1_ESM.docx]

Convergent Evolution of Copy Number Alterations in Multi-Centric Hepatocellular Carcinoma

Carolin Lackner^1@^, Luca Quagliata^2@^, William Cross^3@^, Sebastian Ribi^2^, Karl Heinimann^4^, Viola Paradiso^2^, Cristina Quintavalle^2^, Monika Kovacova^5^, Daniel Baumhoer^2^, Salvatore Piscuoglio^2,6^, Luigi Terracciano^2*^ and Michal Kovac^2*^

1: Institute of Pathology, Auenbruggerplatz 25, 8036 Graz, Austria

2: Institute of Pathology, University Hospital Basel and University of Basel, Schoenbeinstrasse 40, 4056 Basel, Switzerland

3: Department of Tumour Biology, Barts Cancer Institute, Queen Mary University London, UK

4: Medical Genetics and Research Group Human Genomics, University Hospital Basel and University of Basel, Schoenbeinstrasse 40, 4031 Basel, Switzerland

5: The Institute of Mathematics and Physics, Faculty of Mechanical Engineering, Slovak University of Technology, 84248 Bratislava, Slovak Republic

6: Visceral Surgery Research Laboratory, Clarunis, Department of Biomedicine, University of Basel, Basel, Switzerland

@ Equally contributed.

* Correspondence should be addressed to Michal Kovac, [michal.kovac@usb.ch](mailto:michal.kovac@usb.ch) or Luigi Terracciano, [luigi.terracciano@usb.ch](mailto:luigi.terracciano@usb.ch), Institute of Pathology, University Hospital Basel and University of Basel, Schoenbeinstrasse 40, 4031 Basel, Switzerland, Tel.+41 61 328 78 49

Supplementary Tables

Supplementary Table 1: Exome coverage statistics

| **Sample** | **Total Bases Sequenced** | **Mean** | **Third Quartile** | **Median** | **First Quartile** | **% Bases Above 15x** |
| --- | --- | --- | --- | --- | --- | --- |
| P1.CyrL1 | 10876988798 | 171.22 | 206 | 147 | 95 | 93.1 |
| P1.CyrL2 | 9117331296 | 143.528 | 179 | 119 | 71 | 91.7 |
| P1.CyrR1 | 5501953272 | 86.604 | 102 | 73 | 49 | 90.6 |
| P1.CyrR2 | 5400571617 | 85.008 | 102 | 69 | 43 | 89.5 |
| P1.T1.1a (necrotic) | 5643189387 | 88.83 | 104 | 74 | 50 | 91.6 |
| P1.T1.1b (necrotic) | 6263742842 | 98.602 | 115 | 83 | 56 | 91.7 |
| P1.T1.2a (cirrhotic) | 6422246244 | 101.094 | 120 | 85 | 56 | 91.6 |
| P1.T1.2b (cirrhotic) | 4931270131 | 77.63 | 94 | 63 | 39 | 88.5 |
| P1.T2 | 5991796936 | 94.318 | 115 | 81 | 52 | 91 |
| P1.T3.1 | 5487349721 | 86.38 | 102 | 71 | 48 | 90.8 |
| P1.T3.2 | 3720952964 | 58.576 | 71 | 49 | 31 | 85 |
| P2.CyrL1 | 8656462038 | 136.262 | 165 | 115 | 71 | 91.9 |
| P2.CyrR1 | 9399458155 | 147.966 | 176 | 126 | 83 | 92.7 |
| P2.Blood | 10043833115 | 158.102 | 193 | 132 | 81 | 92.5 |
| P2.T1.1 | 9791024711 | 154.126 | 188 | 127 | 80 | 92.6 |
| P2.T1.2 | 9083117606 | 142.982 | 174 | 119 | 74 | 92.2 |
| P2.T2.1 | 8390234651 | 132.076 | 162 | 108 | 66 | 91.5 |
| P2.T2.2 | 8523591674 | 134.176 | 161 | 109 | 67 | 91.6 |
| P2.T3.1 | 9681923562 | 152.404 | 186 | 127 | 78 | 92.3 |
| P2.T3.2 | 8862402375 | 139.51 | 171 | 115 | 70 | 91.7 |

Supplementary Table 2: Somatic SNVs: summary statistics

| **Sample** | **# Variants** | **Mean # of Reads Supporting the Alternative Allele** | **Mean Coverage at Variant Sites** | **VAF** | | | |
| --- | --- | --- | --- | --- | --- | --- | --- |
|  |  |  |  | **Mean** | **Median** | **IQR** | **Max** |
| P1.T1.1 | 11 | 4.83 | 60.60 | 0.12 | 0.10 | 0.07 | 0.33 |
| P1.T1.2 | 10 | 4.50 | 60.33 | 0.11 | 0.14 | 0.09 | 0.23 |
| P1.T2 | 86 | 18.10 | 62.22 | 0.29 | 0.28 | 0.16 | 0.66 |
| P1.T3.1 | 40 | 13.76 | 62.57 | 0.23 | 0.21 | 0.16 | 0.47 |
| P1.T3.2 | 38 | 6.97 | 41.67 | 0.18 | 0.16 | 0.10 | 0.36 |
| P2.T1.1 | 112 | 16.82 | 160.06 | 0.11 | 0.10 | 0.06 | 0.28 |
| P2.T1.2 | 121 | 25.23 | 134.45 | 0.19 | 0.18 | 0.06 | 0.28 |
| P2.T2.1 | 113 | 16.76 | 132.16 | 0.16 | 0.14 | 0.08 | 1.00 |
| P2.T2.2 | 113 | 17.70 | 133.27 | 0.17 | 0.15 | 0.12 | 1.00 |
| P2.T3.1 | 93 | 28.77 | 138.57 | 0.22 | 0.21 | 0.14 | 1.00 |
| P2.T3.2 | 85 | 15.92 | 156.26 | 0.12 | 0.10 | 0.07 | 0.44 |

Supplementary Table 3: Recall rates of somatic variants across multiple tumor regions

| **Sample** | **# Variants** | **# of Re-called Variants** | **# of Not-Recalled Variants** | **Recall Rate** |
| --- | --- | --- | --- | --- |
|  |  |  |  |  |
| P1.T1.1 | 11 | 6 | 5 | 54.5% |
| P1.T1.2 | 10 | 6 | 4 | 60.0% |
| P1.T2 | 86 | NA | NA | NA |
| P1.T3.1 | 40 | 29 | 11 | 72.5% |
| P1.T3.2 | 38 | 29 | 9 | 76.3% |
| P2.T1.1 | 112 | 108 | 4 | 96.4% |
| P2.T1.2 | 121 | 108 | 13 | 89.2% |
| P2.T2.1 | 113 | 87 | 26 | 76.9% |
| P2.T2.2 | 113 | 87 | 26 | 76.9% |
| P2.T3.1 | 93 | 76 | 17 | 81.7% |
| P2.T3.2 | 85 | 76 | 9 | 89.4% |

Supplementary Table 4a: Recalled somatic SNVs: summary statistics

| **Sample** | **# Variants** | **Mean # of Reads Supporting the Alternative Allele** | **Mean Coverage at Variant Sites** | **VAF** | | | |
| --- | --- | --- | --- | --- | --- | --- | --- |
|  |  |  |  | **Mean** | **Median** | **IQR** | **Max** |
| P1.T1.1 | 6 | 8.1 | 95.1 | 0.08 | 0.06 | 0.07 | 0.20 |
| P1.T1.2 | 6 | 4.8 | 89.4 | 0.07 | 0.06 | 0.06 | 0.15 |
| P1.T2 | NA | NA | NA | NA | NA | NA | NA |
| P1.T3.1 | 29 | 16.82 | 68.67 | 0.24 | 0.21 | 0.18 | 0.47 |
| P1.T3.2 | 29 | 7.87 | 45.48 | 0.17 | 0.16 | 0.10 | 0.36 |
| P2.T1.1 | 108 | 17.20 | 163.58 | 0.19 | 0.10 | 0.05 | 0.23 |
| P2.T1.2 | 108 | 26.35 | 140.88 | 0.19 | 0.18 | 0.08 | 0.46 |
| P2.T2.1 | 87 | 19.04 | 148.91 | 0.14 | 0.13 | 0.08 | 0.66 |
| P2.T2.2 | 87 | 20.43 | 149.19 | 0.15 | 0.13 | 0.10 | 0.41 |
| P2.T3.1 | 76 | 32.68 | 153.80 | 0.21 | 0.21 | 0.13 | 0.48 |
| P2.T3.2 | 76 | 16.96 | 165.02 | 0.10 | 0.10 | 0.06 | 0.44 |

Supplementary Table 4b: Non-recalled somatic SNVs: summary statistics

| **Sample** | **# Variants** | **Mean # of Reads Supporting the Alternative Allele** | **Mean Coverage at Variant Sites** | **VAF** | | | |
| --- | --- | --- | --- | --- | --- | --- | --- |
|  |  |  |  | **Mean** | **Median** | **IQR** | **Max** |
| P1.T1.1 | 4 | 4.50 | 33.00 | 0.16 | 0.15 | 0.06 | 0.27 |
| P1.T1.2 | 5 | 3.50 | 23.50 | 0.15 | 0.15 | 0.005 | 0.36 |
| P1.T2 | NA | NA | NA | NA | NA | NA | NA |
| P1.T3.1 | 11 | 5.80 | 48.90 | 0.19 | 0.17 | 0.11 | 0.36 |
| P1.T3.2 | 9 | 4.12 | 20.43 | 0.21 | 0.21 | 0.10 | 0.33 |
| P2.T1.1 | 4 | 4.0 | 28.50 | 0.16 | 0.15 | 0.10 | 0.28 |
| P2.T1.2 | 13 | 17.12 | 86.37 | 0.23 | 0.16 | 0.12 | 0.50 |
| P2.T2.1 | 26 | 9.18 | 71.04 | 0.26 | 0.18 | 0.14 | 1.00 |
| P2.T2.2 | 26 | 8.64 | 77.04 | 0.27 | 0.22 | 0.15 | 1.00 |
| P2.T3.1 | 17 | 9.56 | 62.90 | 0.31 | 0.23 | 0.15 | 1.00 |
| P2.T3.2 | 9 | 7.85 | 90.93 | 0.31 | 0.28 | 0.15 | 0.40 |

Supplementary Table 5: Germline SNVs: summary statistics

| **Sample** | **# Variants** | **Mean # of Reads Supporting the Alternative Allele** | **Mean Coverage at Variant Sites** | **VAF** | | | |
| --- | --- | --- | --- | --- | --- | --- | --- |
|  |  |  |  | **Mean** | **Median** | **IQR** | **Max** |
| P1.CyrL1 | 16032 | 94.47 | 138.70 | 0.68 | 0.54 | 0.52 | 1.00 |
| P1.CyrL2 | 16032 | 111.61 | 162.25 | 0.69 | 0.54 | 0.52 | 1.00 |
| P1.CyrR1 | 16032 | 55.54 | 81.25 | 0.68 | 0.56 | 0.52 | 1.00 |
| P1.CyrR2 | 16032 | 54.50 | 79.70 | 0.68 | 0.56 | 0.53 | 1.00 |
| P1.T1.1 | 16028 | 57.36 | 83.98 | 0.68 | 0.55 | 0.53 | 1.00 |
| P1.T1.2 | 16021 | 64.86 | 94.86 | 0.68 | 0.55 | 0.53 | 1.00 |
| P1.T2 | 16017 | 61.26 | 90.30 | 0.68 | 0.58 | 0.53 | 1.00 |
| P1.T3.1 | 16029 | 55.65 | 81.45 | 0.68 | 0.57 | 0.53 | 1.00 |
| P1.T3.2 | 16020 | 37.72 | 55.19 | 0.68 | 0.58 | 0.53 | 1.00 |
| P2.CyrL1 | 16277 | 103.69 | 150.67 | 0.69 | 0.55 | 0.52 | 1.00 |
| P2.CyrR1 | 16277 | 88.46 | 128.45 | 0.69 | 0.55 | 0.55 | 1.00 |
| P2.Blood | 16277 | 96.31 | 139.67 | 0.69 | 0.55 | 0.52 | 1.00 |
| P2.T1.1 | 16273 | 100.91 | 146.799 | 0.69 | 0.55 | 0.52 | 1.00 |
| P2.T1.2 | 16265 | 92.97 | 135.21 | 0.69 | 0.56 | 0.52 | 1.00 |
| P2.T2.1 | 16264 | 85.58 | 124.54 | 0.69 | 0.56 | 0.52 | 1.00 |
| P2.T2.2 | 16271 | 86.18 | 125.28 | 0.69 | 0.56 | 0.52 | 1.00 |
| P2.T3.1 | 16272 | 90.38 | 131.48 | 0.69 | 0.55 | 0.52 | 1.00 |
| P2.T3.2 | 16274 | 99.31 | 144.25 | 0.69 | 0.55 | 0.52 | 1.00 |

Supplementary Table 6: Recall rates of germline variants across multiple tumor regions

| **Sample** | **# Variants** | **# of Re-called Variants** | **# of Not-Recalled Variants** | **Recall Rate** |
| --- | --- | --- | --- | --- |
|  |  |  |  |  |
| P1.T1.1 | 16028 | 16032 | 4 | >99.99% |
| P1.T1.2 | 16021 | 16032 | 11 | >99.99% |
| P1.T2 | 16017 | 16032 | 15 | >99.99% |
| P1.T3.1 | 16029 | 16032 | 3 | >99.99% |
| P1.T3.2 | 16020 | 16032 | 12 | >99.99% |
| P2.T1.1 | 16273 | 16277 | 4 | >99.99% |
| P2.T1.2 | 16265 | 16277 | 11 | >99.99% |
| P2.T2.1 | 16264 | 16277 | 12 | >99.99% |
| P2.T2.2 | 16271 | 16277 | 6 | >99.99% |
| P2.T3.1 | 16272 | 16277 | 5 | >99.99% |
| P2.T3.2 | 16274 | 16277 | 3 | >99.99% |

Supplementary Table 7: Somatic mutations that passed technical replication.

| **Samples** | **Gene** | **Variant Annotation** | **VAF** | **In Both Regions?** |
| --- | --- | --- | --- | --- |
|  |  |  |  |  |
| P1.T1 | FANCD2 | NM_033084:exon42:c.C4061G:p.T1354S | 0.09 | No |
| P1.T2 | TLX1 | NM_005521:exon2:c.C704A:p.A235D | 0.25 | NA |
| P1.T2 | ATM | NM_000051:exon19:c.T2909C:p.L970P | 0.34 | NA |
| P1.T2 | RAD50 | NM_005732:exon9:c.A1303T:p.K435X | 0.47 | NA |
| P1.T2 | TRIP11 | NM_004239:exon16:c.A5244T:p.E1748D | 0.14 | NA |
| P1.T2 | DNMT3A | NM_175629:exon5:c.A456C:p.E152D | 0.22 | NA |
| P1.T3 | CDH1 | NM_004360:exon3:c.A226C:p.K76Q | 0.39, 0.45 | Yes |
| P2.T2 | CTNNB1 | NM_001904:exon3:c.G100C:p.G34R | 0.07, 0.13 | Yes |
| P2.T1 | TP53 | NM_000546:exon10:c.994-1G>T | 0.10, 0.48 | Yes |
| P2.T1 | HSP90AB1 | NM_007355:exon12:c.G2156A:p.R719H | 0.06, 0.12 | Yes |
| P2.T1 | LRP1B | NM_018557:exon85:c.C13060A:p.P4354T | 0.16, 0.21 | Yes |
| P2.T1 | MBD1 | NM_015846:exon11:c.G1060A:p.D354N  NM_015845:exon10:c.G991A:p.D331N  NM_015847:exon10:c.G913A:p.D305N | 0.03, 0.46 | Yes |
| P2.T2 | TP53 | NM_000546:exon6:c.G587T:p.R196L | 0.11, 0.11 | Yes |
| P2.T2 | ETV4 | NM_001986:exon13:c.C1298G:p.P433R | 0.20, 0.37 | Yes |
| P2.T2 | FN1 | NM_002026:exon26:c.4069+1G>T | 0.26, 0.17 | Yes |
| P2.T2 | TNK2 | NM_005781:exon8:c.G1087A:p.V363I | 0.20, 0.00 | No |
| P2.T2 | EZH2 | NM_004456:exon11:c.T1357C:p.Y453H | 0.03, 0.13 | Yes |
| P2.T3 | FLT3 | NM_004119:exon15:c.T1847A:p.L616Q | 0.08, 0.05 | Yes |
| P2.T3 | NFE2L2 | NM_006164:exon4:c.C503A:p.S168Y | 0.28, 0.08 | Yes |
| P2.T3 | TBX22 | NM_016954:exon2:c.A239G:p.Y80C | 0.21, 0.05 | Yes |
| P2.T3 | PAX7 | NM_013945:exon8:c.C1299A:p.S433R | 0.33, 0.15 | Yes |

Supplementary Table 8: Mean variant allele frequencies (VAF) of non/recalled mutations.

|  | **Non-recalled**  **Variants** | **Recalled**  **Variants** | **Two-tailed P Value** |
| --- | --- | --- | --- |
| **Patient 1** | 0.17 | 0.14 | 0.4313 |
| **Patient 2** | 0.25 | 0.16 | 0.0093 |

Supplementary Table 9: Cancer driver mutations

|  | **Gene** | **Variant Description** | **CMCC Class*** |
| --- | --- | --- | --- |
| **P1.T2** | TERT | g.5:1295228G>A (promoter) | 5 |
| **P2.T2** | TERT | g.5:1295228G>A (promoter) | 5 |
| **P2.T3** | TERT | g.5:1295228G>A (promoter) | 5 |
| **P1.T2** | CDK1A | p.K16fs | 5 |
| **P1.T2** | KMT2D | p.E731X | 5 |
| **P1.T2** | RAD50 | p.K435X | 5 |
| **P1.T3** | CDH1 | p.K76Q | 3 |
| **P2.T1** | TP53 | c.994-1G>T | 5 |
| **P2.T2** | TP53 | p.R196L | 5 |
| **P2.T2** | CTNNB1 | p.G34R | 5 |
